# Case report: A novel frameshift mutation in BRSK2 causes autism in a 16-year old Chinese boy

**DOI:** 10.3389/fpsyt.2023.1205204

**Published:** 2023-08-21

**Authors:** Yu Hu, Miao Li, Yanmei Shen, Tianyun Wang, Qiwei Liu, Zhonghua Lu, Hong Wang, Xuerong Luo, Lixin Yang

**Affiliations:** ^1^The Brain Cognition and Brain Disease Institute, Shenzhen Institute of Advanced Technology, Chinese Academy of Sciences, Shenzhen, China; ^2^Shenzhen-Hong Kong Institute of Brain Science-Shenzhen Fundamental Research Institutions, Shenzhen, China; ^3^Department of Psychiatry, National Clinical Research Center for Mental Disorders, The Second Xiangya Hospital of Central South University, Changsha, Hunan, China; ^4^Department of Medical Genetics, Center for Medical Genetics, School of Basic Medical Sciences, Peking University Health Science Center, Beijing, China; ^5^Neuroscience Research Institute, Peking University, Beijing, China; ^6^Key Laboratory for Neuroscience, Ministry of Education of China and National Health Commission of China, Beijing, China; ^7^Autism Research Center, Peking University Health Science Center, Beijing, China; ^8^State Key Laboratory of Primate Biomedical Research, Institute of Primate Translational Medicine, Kunming University of Science and Technology, Kunming, China

**Keywords:** BRSK2, autism, *de novo* mutation, exome sequence, clinical phenotype

## Abstract

Serine/threonine protein kinases are involved in axon formation and neuronal polarization and have recently been implicated in autism spectrum disorder (ASD) and neurodevelopmental disorders (NDD). Here, we focus on BRSK2, which encodes brain-specific serine/threonine protein kinase 2. Although previous studies have reported 19 unrelated patients with BRSK2 pathogenic variation, only 15 of 19 patients have detailed clinical data. Therefore, more case reports are needed to enrich the phenotype associated with BRSK2 mutations. In this study, we report a novel *de novo* frameshift variant (c.442del, p.L148Cfs^*^39) identified by exome sequencing in a 16 year-old Chinese boy with ASD. The proband presented with attention-deficit, auditory hallucinations, limb tremor, and abnormal brain electrical activity mapping. This study expands the phenotypic spectrum of BRSK2-related cases and reveals the highly variable severity of disorders associated with BRSK2.

## Introduction

Children with autism spectrum disorder (ASD, also known as autism) share some symptoms, such as differences in social communication, and stereotyped, repetitive, or restricted behaviors or interests, based on the Diagnostic and Statistical Manual of Mental Disorders, Fifth Edition, Text Revision (DSM-5-TR) (1). The male-to-female ratio of patients with ASD is 4.2 (2). In addition, the prevalence of autism continues to increase, with serious implications for affected families and society.

Autism has a strong and diverse genetic background. Recent large cohort studies revealed that *BRSK2* has strong statistical support and is a genome-wide significant risk gene for ASD (3). Brain selective kinase 2 (BRSK2) is a serine/threonine protein kinase that belongs to the AMPK-related protein kinase family, which also includes BRSK1 and 11 other kinases (4). BRSK2 was found to be selectively expressed in the mouse brain, and exhibited the highest expression in the brain among various human organs (5). Recent studies using KO mice suggested that BRSK1 and BRSK2 are essential for the development of polarity of forebrain neurons, which realizes distinct properties of axons and dendrites (6). In addition, BRSK2 mutations have been reported to be associated with ASD and neurodevelopmental disorders (NDD) (3). However, detailed clinical courses have not been described in many cases of BRSK2 mutations.

In this study, clinical exome sequencing was performed on an ASD patient and his family members, and the results revealed the presence of c.442del, p.L148Cfs^*^39, which is a *de novo* BRSK2 pathogenic mutation, in this proband. Notably, the patient exhibited acousma and abnormal EEG. This study expands the phenotypic spectrum associated with BRSK2 mutations.

## Materials and methods

### Psychological assessment

The Autism Diagnostic Observation Scale-Second Edition (ADOS) was used in autism clinical judgment. This scale is one of the most frequently used research tools, which has a standardized structure (7). Scale for assessment of negative symptoms (SANS) has 24 items which was used to measure the severity of negative symptoms in schizophrenia. Its score ranges between 0-120. Scale for assessment of positive symptoms (SAPS) has 35 items and its score ranges between 0 and 165 (8). The Wechsler Intelligence Scale for Children-Fifth Edition (WISC-V) is a valuable IQ test tool for assessing cognitive abilities in children between the ages of 6 and 16 years old (9).

### EEG recording and data analysis

The EEG recording were obtained with 16-electrode Stellate Harmonie EEG systems (Natus Medical Incorporated). The EEG signals were preprocessed using a 0.1–100 Hz band-pass filter and the data was analyzed using Harmonie software (Stellate HARMONIR 7.0).

### Sample preparation and DNA extraction

Peripheral blood of the proband and his family members was sampled by using EDTA tubes at The Second Xiangya Hospital of Central South University. DNA extraction was achieved by utilizing a DNA Blood Midi/Mini kit (Qiagen, Germany), and DNA concentrations were measured by utilizing a DNA Assay Kit (Qubit^®^, Life Technologies, USA).

### Exome sequencing and variant prioritization

Exome sequencing of genomic DNA samples of the patient and his family members was executed using the Nonaseq 6000 platform (Illumina, USA). An exome library was developed by utilizing xGen Exome Research Panel V1.0 (Integrated DNA Technologies, USA). The raw paired-end reads were aligned to hg38/GRCh38, which serves as a reference genome, with BWA Enrichment. The Genome Analysis Tool Kit (GATK) was used to call variants. ANNOVAR was employed for annotation of Variant Call Format (VCF) acquired previously. The Human Genome Mutation Database (HGMD) and 1,000 Genomes Project were applied to characterize the detected variants. All variants were categorized based on mutation, location, and frequency. The threshold of low frequency filter was minor allele frequency (MAF) < 0.05. The synonymous SNVs and unannotated variants were discarded, and only SNVs observed in splice sites or exons were further investigated. Missense variants were predicted by utilizing the bioinformatics mutation prediction software programs (SIFT). The variations were categorized into groups of benign, likely benign, uncertain significance, likely pathogenic, and pathogenic by using American College of Medical Genetics and Genomics (ACMG). The AlphaFold tool was used to model and visualize the mutant and wild-type protein structures.

### Sanger sequencing

The BRSK2 mutation was confirmed by Sanger sequencing of exon5, as well as its flanking intron regions (NM_001256627.2) of the proband and his family members. DNA amplification was achieved by utilizing PCR with gene-specific primers, and purification of the PCR products was achieved by utilizing a PCR Purification Kit (Qiagen, Germany). Additionally, Sanger sequencing using the ABI 3730xl DNA Analyzer (Applied Biosystems, USA) was executed on the purified PCR products to confirm BRSK2 mutation, and the results were investigated by utilizing SnapGene V.4.1.9 (SnapGene, USA).

## Results

### Clinical description

**Figure 1:**
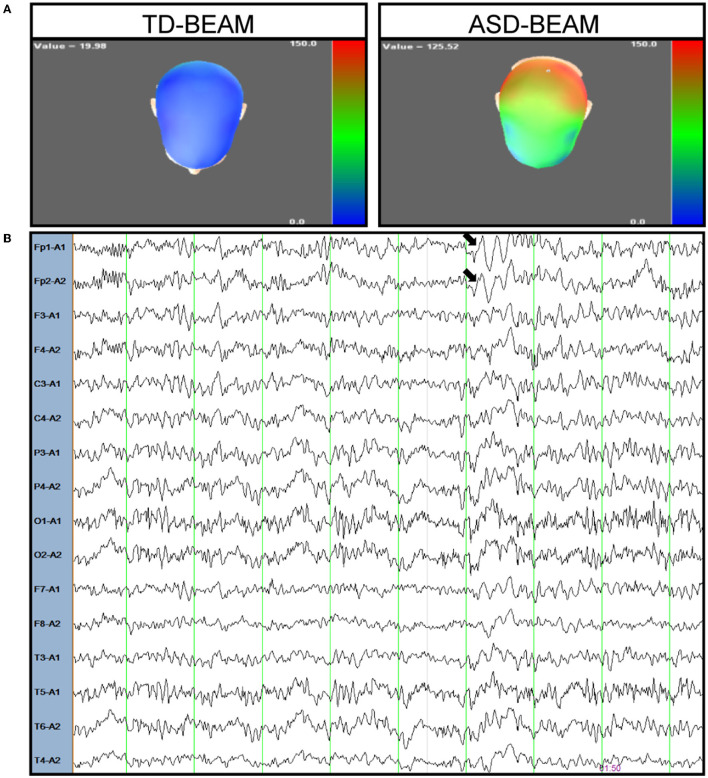
Electroencephalogram of the patient. **(A)** The power of theta band was observed increased in the patient (right) compared to a typical developing boy (left), colors depicted represent power (μV2) within theta bands across regions. Blue areas correspond to the lower power for theta band and the red areas correspond to higher power. **(B)** A representative EEG trace recorded from the patient during resting condition. The arrow marks the increase in the theta band in frontal brain region.

The patient was a 16-year-old boy who is the first child of a healthy, non-consanguineous couple. There was no family history of neurodevelopmental disorders. He was born at 39 weeks gestation with the following auxological parameters: length 50 cm and weight 3,900 g. The mother reported that the proband began crawling at 8 months, walking independently at the age of 1 year, saying single words at the age of 2 years, and saying simple sentences at 30 months. Meanwhile, eye contact with the patient exhibited no problems, and sphincter control was obtained at 24 months. At the age of 5 years, the proband was still unable to perform fine motor tasks, such as tying shoelaces, but interaction with peers was normal. The patient entered school at the age of 6, but exhibited severe difficulties in learning Chinese and inattention. The mother reported that the boy had auditory hallucinations and giggled involuntarily at the age of 11. The boy told his mother that he heard a male teacher talking to him and telling him to do things. This patient was first diagnosed with mental retardation in the Children's hospital in Tianjin at the age of 11. After 2 years of functional training and rehabilitation exercises, there was no significant improvement. However, the patient was found to have limb tremors at the age of 13. Although the proband did not have any dietary changes, he had a sleep disorder. He was too agitated to fall asleep for 2 days every month. The Wechsler Intelligence Scale for Children, Fifth Edition (WISC-V) was used to test the patient's level of intellectual functions. His full-scale intelligence quotient (FSIQ) score was 38, which was below 70, and he was considered to have moderate intellectual disability. The Autism Diagnostic Observation Schedule (ADOS) score and the autism cut-off score of the proband were 18 and 10, respectively. The scale for the Assessment of Positive Symptoms (SAPS) and the scale for the Assessment of Negative Symptoms (SANS) were also used to assess the patient's schizophrenia symptoms. The scores of SAPS and SANS were 10 and 40, respectively, both below the cut-off score of 50. Based on these assessment results, the boy was second diagnosed with autistic spectrum disorder at Xiangya Hospital at the age of 13. The MRI results of his brain were normal, while the EEG results showed abnormal changes in brain electrical activity mapping (BEAM). The power of the theta band increased in the patient compared to typically developing (TD) boy of the same age (see Figure 1). Although the BEAM of this patient was altered, his mother reported that he never had a seizure.

Through literature review, we identified 19 cases with BRSK2 pathogenic mutations. Fifteen of the 19 cases reported partial clinical data. All cases with partial clinincal data were diagnosed with autism and presented with speech delay (100%, 15/15). Three of the 15 cases were female, and their age ranged from 3 to 19 years at first report. Twelve of the 15 cases presented with motor delay (80%, 12/15). In addition, some were reported to have sleep disorders (20%, 3/15), feeding problems (13.34%, 2/15), epilepsy (13.34%, 2/15), and schizophrenia (6.67%, 1/15). Details are presented in Supplementary Table S1.

### Genetic analysis

**Figure 2:**
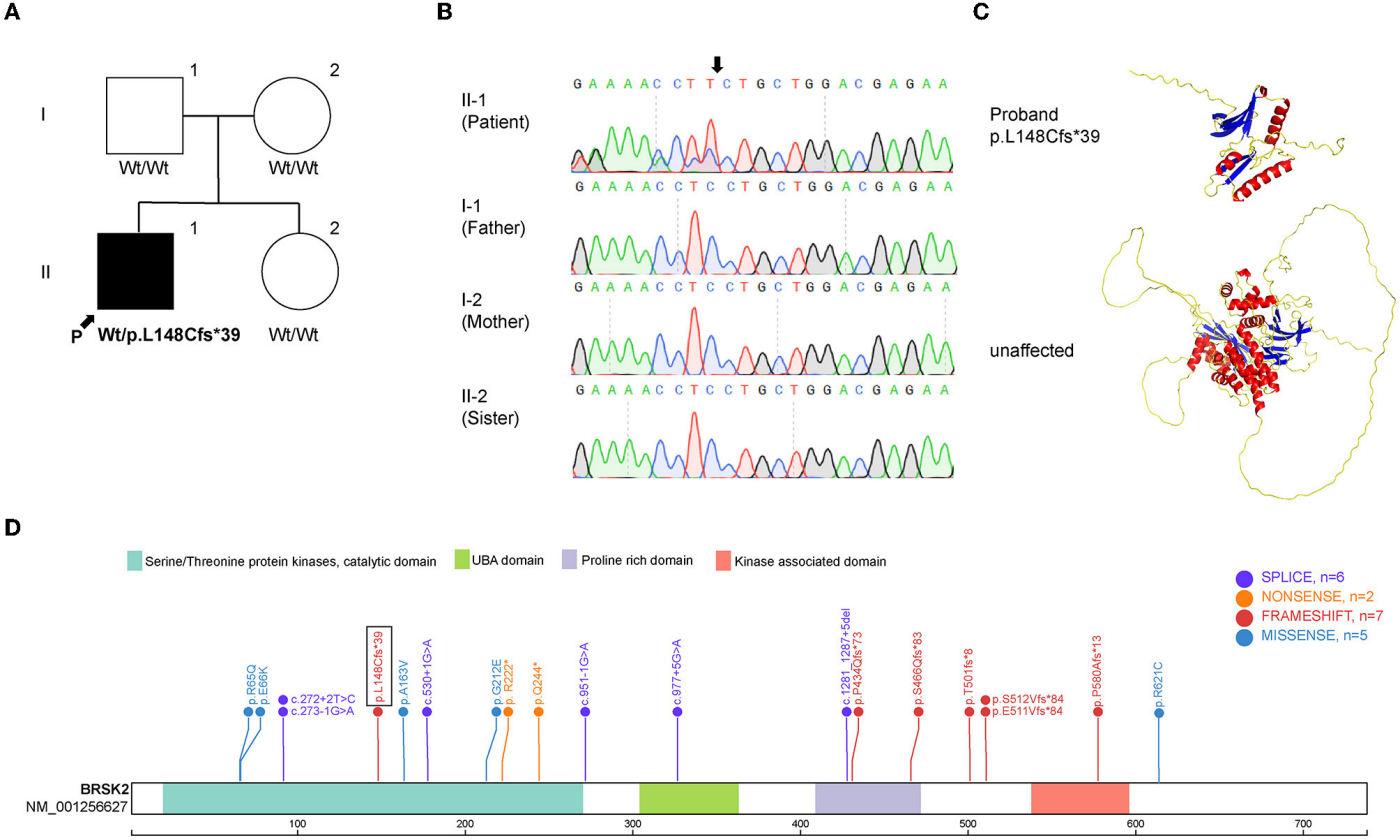
Mutation analysis of BRSK2. **(A)** Pedigree of the family described in this report. **(B)** Sequences around the BRSK2 mutation in the patient and his family. The patient carries a C deletion, which leads to a loss function variant, and his family carrier normal sequence. **(C)** The computational modeling of wild type and mutant BRSK2 in human. **(D)** Genomic structure of BRSK2 mutations reported previously are shown and the mutation found in this study is shown in a box.

Exome sequencing was performed on the proband and his family members. The mean depth of coverage was 20X. The mapping rates of all samples exceeded 98%. The analysis revealed the presence of c.442del, p.L148Cfs^*^39, which is a *de novo* frameshift variant in exon 5 of BRSK2 gene on chromosome 11 (Figure 2A). Sanger sequencing demonstrated that the heterozygous variant was present in the proband, but not in his parents or his sister (Figure 2B). This C-deletion mutation in BRSK2 leads to a premature translation termination codon and a 187 amino acid truncated protein (Figure 2C). This *de novo* frameshift deletion was identified for the first time in this study and is not present in the SPARK or SFARI gene databases (Figure 2D) (one sequence deletion, two non-sense variants, six frameshift variants, six missense variants, and six splice-site variants).

## Discussion

This study reports a novel pathogenic BRSK2 variant in an ASD patient. The proband presented with speech delays, attention-deficit, and acousma. Exome sequencing demonstrated the presence of c.442del, p.L148Cfs^*^39, which is a *de novo* frameshift variant predicted to be deleterious. Previous publications have reported 19 non-sense, splice alteration, frameshift, and deleterious missense variations in BRSK2. These mutations were likely responsible for the phenotypes of these patients with or without ASD (3, 10–16). We compared the reported clinical phenotypes caused by the mutations located in the same catalytic domain. There were four missense mutations, three splice alterations, two non-sense mutations and one single-base deletion in the same domain. Three of the four missense mutations were all G to A variations, but they caused different symptoms, including intermittent horizontal nystagmus, sleep disorder and undescended testis. One splice alteration caused mild gait ataxia and tremor in a female patient. This symptom is similar to the limb tremor of the patient in our study. Although facial features have been reported in some probands, no consistent set of features was observed in our case. Previous publications indicated the heterogeneity among different mutation sites. Hence, more detailed case reports are necessary to expand the phenotypic spectrum associated with BRSK2 mutations. In this study, abnormal brain electrical activity mapping and acousma were reported for the first time in an ASD patient with BRSK2 mutation. Although the patient's theta band power was altered, he never had seizures. In previous studies, Pablo Billeke and his group assessed the electroencephalographic activity of ASD and TD subjects during a working memory task. They found that impaired theta modulation correlated with autistic symptoms (17), which is consistent with our findings. It has been suggested that the alteration of the theta band may be related to the physiopathology of ASD.

BRSK2, also known as SAD-A, is located at 11p15.5 and encodes 736 amino acids. The protein comprises multiple domains, including a proline-rich domain (aa 424-468), a kinase-associated domain (KA1; aa 530-653), a protein kinase (aa 19-270), and a ubiquitin-associated domain (UBA; aa 297-339) (18). BRSK2 is highly conserved in evolution and exclusively expressed in the vertebrate brain (14). In fact, BRSK2 is involved in axonogenesis and cortical neuron polarization (4, 6, 19, 20). Previous studies have reported that BRSK2 interacts with NDD-associated genes such as autism and developmental delay (DD) and/or intellectual disability (ID). BRSK2 can phosphorylate TSC2 and suppress mTORC1 activity (21, 22). As a component of the TSC signaling pathway, TSC2 regulates cell size and growth. The TSC signaling pathway is associated with autophagy during early axonal growth. Also, BRSK2 interacts with PTEN, which is associated with various developmental disorders (e.g., autism). PTEN knockout mice exhibit neuronal structure malformation and autistic features caused by aberrant TSC-mTORC1 signaling (23, 24). A previous publication reported that single mutant BRSK1 or BRSK2 mice were healthy and fertile, but BRSK1 and BRSK2 double knockout mice showed perinatal lethality with a severe defects in axon differentiation and died within 2 h after birth (4). Conversely, another study demonstrated that on a C57BL/6N background, BRSK2 is essential for cortical development. The BRKS2 knockout mice died within a few days after birth (10). Meanwhile, BRSK2-mutant zebrafish exhibited ASD-like features (e.g., developmental delay, social impairment) (4, 14).

BRSK1, also known as SAD-B, is the homolog of BRSK2. BRSK1 acts as a multifunctional regulator, by phosphorylating its downstream proteins it is involved in many biological processes. BRSK1 can phosphorylateγ-tubulin to regulate centrosome duplication, and phosphorylate CAST to control active zone vesicle recycling for synaptic depression (25, 26). Furthermore, BRSK1 knockout mice showed impaired contextual fear learning. BRSK1 plays a critical role in controlling vesicle release properties and regulating hippocampal function in the mature brain (27). However, when we queried the SFARI Gene database, which tracks the ever-expanding genetic risk factors of autism, surprisingly, we found that BRSK1 has not yet been included among the autism risk genes. Although the evidence suggests that BRSK1 and BRSK2 play a key roles in cortical and neurodevelopmental processes, the functional compensation between BRSK1 and BRSK2 has not been sufficiently studied. Hence, more work is needed to investigate the function of BRSK1 and BRSK2.

In conclusion, we report a pathogenic *de novo* BRSK2 mutation in an ASD patient, and our findings expand the phenotypic spectrum associated with BRSK2 mutations.

## Data availability statement

The datasets for this article are not publicly available due to concerns regarding participant/patient anonymity. Requests to access the datasets should be directed to the corresponding authors.

## Ethics statement

The studies involving human participants were reviewed and approved by Institutional Review Board, Shenzhen Institute of Advanced Technology, Chinese Academy of Sciences. Written informed consent to participate in this study was provided by the participants' legal guardian/next of kin. Written informed consent was obtained from the individual(s), and minor(s)' legal guardian/next of kin, for the publication of any potentially identifiable images or data included in this article.

## Author contributions

LY and TW developed the writing plan and drafted the manuscript. XL and YS performed the autism clinical evaluation. YH, ML, HW, and QL performed the experiments. ZL, LY, and QL developed the figures. LY reviewed and approved the final manuscript. All authors have read and agreed to published version of the manuscript.
